# Cumulative Family Risk and Migrant Children’s School Adjustment: A Moderated Mediation Model of Relative Deprivation and Beliefs About Adversity

**DOI:** 10.3390/bs15121690

**Published:** 2025-12-06

**Authors:** Shuying Fu, Zhicao Zhang, Qinqiu Gao

**Affiliations:** 1 Faculty of Psychology, Tianjin Normal University, Tianjin 300387, China; gaoqq@stu.tjnu.edu.cn; 2 Jiangsu Provincial University Key Laboratory of Child Cognitive Development and Mental Health, Yancheng Teachers University, Yancheng 224002, China; zhang_zc1976@163.com; 3 College of Teacher Education, Taishan University, Taian 271000, China

**Keywords:** migrant children, cumulative family risk, school adjustment, relative deprivation, beliefs about adversity

## Abstract

Migrant children encounter numerous survival challenges during their development, which may contribute to difficulties they may face in adapting to new school environments. Although existing research has confirmed that multiple risk factors within the family microsystem negatively affect these children’s school adjustment, the cumulative impact of these risks and their underlying mechanisms remain insufficiently explored. A total of 2498 students participated in this study, including 1370 non-migrant children and 1128 migrant children (mean age = 12.83 ± 1.21 years; 576 boys), recruited from three middle schools in Jiangsu Province. The results show that (1) cumulative family risk had a significant negative predictive effect on school adjustment; (2) relative deprivation played a partial mediating role in this relationship; and (3) beliefs about adversity moderated the latter half of the mediation pathway, serving a protective function. Exposure to multiple family risk factors may heighten migrant children’s relative deprivation, thereby adversely influencing their school adjustment—a pathway further moderated by adversity beliefs. The present study not only contributes to a deeper theoretical understanding of the link between cumulative family risk and school adjustment, and the underlying mechanisms thereof, but also offers practical insights that can help design interventions that aim to enhance school adjustment among migrant children.

## 1. Introduction

Migrant children (MC) are defined as those who have moved from rural areas to urban centers and have resided with their parents in these cities for a minimum of six months (Han et al., 2020). According to the 2020 census in China, there are approximately 71.09 million migrant children across the country, with 51.55 million of them being of school age (from primary to high school), which accounts for 17.32% of the total child population in the nation (Zhai & Liu, 2021). For these children, schools serve not only as places for learning but also as essential environments for social integration into urban life. Adapting to new educational environments often presents two main challenges: forming new social connections within the school (relationships with teachers and peers) and acclimating to different teaching methods, curricula, and school cultures. This intricate process of adjustment leads to a notably higher risk of maladjustment for migrant children compared to their local counterparts.

School adjustment (SA) encompasses how well students adapt to both the academic and social dimensions of their educational environment, which includes their involvement, developmental growth, and success in their studies (Ladd et al., 1997). Evidence indicates that children from migrant backgrounds encounter more significant obstacles to adjusting to school, especially regarding their academic success and relationships with peers (Gannon et al., 2023; Winsper et al., 2016). These challenges have a profound effect on their overall well-being, with research highlighting that school adjustment is a key indicator of mental health outcomes (Arslan & Allen, 2022; Chen et al., 2022; L. Zhang et al., 2024).

This issue is especially critical during middle school years when adolescents experience rapid physical and psychological changes while adapting to urban environments (Yu et al., 2024). Inadequate school adjustment during this sensitive period may lead to academic struggles, emotional distress, and long-term consequences for social functioning and life satisfaction. The present study investigates school adjustment mechanisms among migrant middle school students to develop effective support strategies.

Ecological systems theory posits that an individual’s growth is influenced by complex interactions with various environmental systems, with the family being the most significant microsystem impacting the psychosocial and behavioral development of migrant children (Wertsch, 2005). Empirical evidence has consistently demonstrated that migrant children’s school adjustment disorders stem from cumulative family risk factors (CFR, Lynn, 2022), particularly parental divorce, financial hardship, and dysfunctional parent–child relationships (Boele et al., 2020; He & Yu, 2022; Tetzner & Becker, 2015). Such adversities elevate children’s vulnerability to maladaptive outcomes (Wertsch, 2005). Nevertheless, existing research has predominantly examined family risks in isolation, while the compounding effects of multiple familial risk factors remain understudied. Notably, children exposed to co-occurring risks have demonstrated significantly higher susceptibility to adjustment disorders compared to those facing single risk factors (Ashworth & Humphrey, 2020; G. Yang et al., 2023).

Empirical studies highlight the crucial importance of examining the cumulative effects of multiple risk factors on migrant children’s school adjustment. Addy et al. found that approximately 41% of children face 1–2 family risk factors, while up to 20% are exposed to 3 or more compounding risks (Jiang et al., 2017). According to the cumulative risk model (Evans et al., 2013), the accumulation of multiple risk factors not only exacerbates harm to individuals but also leads to more enduring negative impacts. Consequently, the total number of risk factors is a more effective predictor of adverse developmental outcomes in children than any single risk factor. Importantly, migrant children, as a particularly vulnerable demographic, experience a significantly higher level of cumulative risk compared to their non-migrant counterparts (Gatt et al., 2020). For instance, Diler et al. (2003) observed that migrant children tend to have parents with lower educational attainment and are subject to greater economic pressure in their family environments. Supporting this, a study by J. Wang et al. (2017) conducted with a Chinese sample found that, compared to non-migrant children, migrant children not only endure higher levels of economic stress in the family but are also more likely to experience parental neglect. This evidence collectively indicates that migrant children often face a pattern of exposure to “multiple overlapping” family risk factors. Nevertheless, research investigating the negative effects of cumulative family risk factors on migrant children’s school adjustment, and the underlying mechanisms involved, remains relatively scarce, underscoring a clear need for further in-depth investigation.

The assessment of cumulative family risk factors typically involves categorizing and aggregating the different elements of family risk (J. Xiong et al., 2023). Operationally, categorical variables are coded as “1” (risk) for a theoretically defined reference group and “0” (no risk) for others, while continuous variables are dichotomized at the 75th percentile of the sample distribution. Although this method has limitations—such as information loss from dichotomizing continuous variables and the absence of risk factor weighting—Evans et al. (2013), in their systematic review of multiple risk modeling approaches spanning four decades, argue that it offers notable advantages. These include more robust parameter estimates due to the lack of weighting, as well as a high level of interpretability, which facilitates communication with both policymakers and the general public. As a result, this computational approach remains the most widely used in the field to date.

The cumulative risk model offers a conceptual basis for analyzing this issue, highlighting that the combined effect of multiple risk factors is a better predictor of children’s development results than individual risks (Evans et al., 2013). In practice, this model utilizes a binary scoring system (1 for risk being present, and 0 for no risk) to create a cumulative risk index via straightforward addition. This streamlined and efficient approach to evaluation has proven to be highly feasible and exhibits strong predictive validity in studies involving at-risk populations (Appleyard et al., 2005; G. Yang et al., 2023).

Relative deprivation (RD) is the personal cognitive and emotional response experienced by individuals or groups who feel they are at a disadvantage in comparison to others, leading to feelings of dissatisfaction and resentment (M. Xiong & Ye, 2016). Studies indicate that factors such as low socioeconomic status and inadequate parental education heighten the risk of experiencing relative deprivation (Y. Wang & Meng, 2024; Ye & Xiong, 2017). For migrant children whose parents are typically rural laborers engaged in unstable, low-paying jobs, this socioeconomic disadvantage fosters strong perceptions of rights deprivation when these children compare themselves to their urban peers (J. Zhang & Tao, 2013).

Relative deprivation theory posits that risk exposure triggers relative deprivation through a perceived loss of benefits, impairing adjustment (Smith et al., 2012; Wickham et al., 2013). Callan et al. (2017) found that relative deprivation mediates the family economic risk-adjustment link, exacerbating the negative effects of economic hardship. Longitudinal findings from Zhang and Tao reveal that the severity of deprivation is a predictor of a decline in adjustment (J. Zhang & Tao, 2013). Consequently, when individuals face multiple simultaneous risk factors, the cumulative impact may significantly amplify the severity of relative deprivation, leading to more enduring negative consequences for their adjustment levels (Ohno et al., 2023).

Notably, many children from migrant backgrounds have shown impressive psychological strength and have successfully adapted, even when confronted with various challenges (Mou et al., 2013). According to cognitive adaptation theory (Taylor, 1983), individuals actively employ psychological strategies to mitigate risks, with beliefs about adversity serving as a crucial protective mechanism in this adaptive process (Shek et al., 2003). Beliefs about adversity (BAs) represent how individuals assess difficulties, including their perceived origins, effects, and coping strategies (Shek, 2005). Those with positive beliefs about adversity tend to view challenges as manageable (“Hardship cultivates growth”) rather than as insurmountable threats, thereby bolstering resilience and adaptability (Shek, 2004).

Studies have shown that having a strong belief in the positive aspects of adversity can protect individuals from the detrimental effects of various risk factors, thereby aiding in their psychological adaptation (Callan et al., 2015; Liu & Wang, 2018; Zhao et al., 2013). Specifically, such beliefs help lessen the negative consequences that socioeconomic challenges, such as financial difficulties and conflicts between parents and children, have on the mental health of adolescents, leading to lower levels of depression and behavioral problems (Li et al., 2024; Luo et al., 2023; B. Yang et al., 2021). Furthermore, they contribute to improved well-being and life satisfaction among children (Shek, 2004; Zhao et al., 2017). For migrant children, these beliefs are particularly crucial as they promote effective cognitive and emotional management. For those encountering multiple challenges, a positive outlook on adversity facilitates a realistic assessment of difficulties while enhancing emotional regulation and overall coping skills.

Previous studies have investigated the connections between various family-related challenges, feelings of relative deprivation, perceptions of hardship, and adjustment outcomes; however, significant gaps remain. Firstly, there has been no comprehensive analysis of how the accumulation of family-related challenges influences migrant children’s adaptation to a new school environment. Most existing research has concentrated on individual risk factors, such as financial difficulties and conflicts between parents and children (Martinez-Escudero et al., 2023; J. Zhang et al., 2023, 2022), overlooking the effects of these factors when considered together. Secondly, while risks associated with the school environment, like negative teacher–student interactions and social rejection, have been extensively researched (Pu et al., 2024; Sun et al., 2020; Zhou et al., 2022), the impact of family-related risks, including poverty and parental separation, on school adjustment is still not well understood.

**Figure 1:**
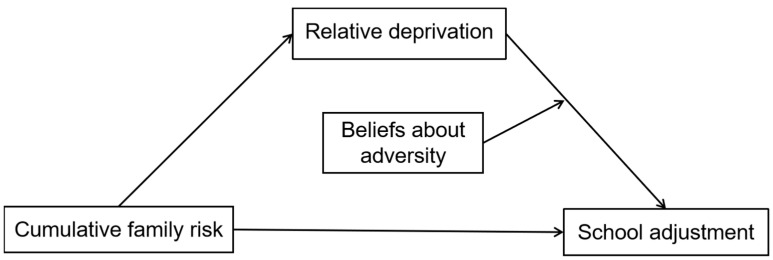
Hypothesized moderated mediation model.

Drawing from the cumulative risk model, relative deprivation theory, and cognitive adaptation theory, this research utilizes a moderated mediation framework to thoroughly investigate the psychological processes by which accumulated family risks affect the school adjustment of migrant children (refer to Figure 1). The research evaluates the following three primary hypotheses:

**H1.**  *Cumulative family risk negatively predicts migrant children’s school adjustment.*

**H2.**  *Relative deprivation mediate the correlation between cumulative family risk and migrant children’s school adjustment.*

**H3.**  *Beliefs about adversity moderate the association between relative deprivation and school adjustment, with a weaker negative effect at higher levels of beliefs about adversity.*

## 2. Materials and Methods

### 2.1. Participants

The research took place in three middle schools situated in the economically thriving coastal cities of Yancheng and Zhenjiang in eastern China, both of which have large populations of migrant children. Out of an initial 2591 questionnaires gathered, 93 were discarded due to incomplete information, leaving 2498 valid responses and a retention rate of 96.41%. The final sample for analysis comprised 1128 migrant children (average age = 12.83 ± 1.21 years), with a nearly equal distribution of genders (576 boys, average age = 13.16 ± 1.12, and 552 girls, average age = 12.48 ± 1.25) and grades (641 in seventh grade and 487 in eighth grade). All research procedures were approved by our institutional review board, and the questionnaires were distributed during regular class sessions with the schools’ consent.

### 2.2. Measures

#### 2.2.1. Cumulative Family Risk

This research examined seven possible family risk factors, assigning a code of “0” to indicate absence and “1” to denote the presence of risk. The specifics of coding and assessment are outlined as follows:

(1) Family structure: Coded “0” when both biological parents are present, and “1” for any other family arrangements.
(2) Educational attainment of parents: Coded “1” if neither parent had finished high school.
(3) Parent–child separation: Designated “1” if one or both parents were away due to work or other migration within the previous six months; the presence of both parents was coded as “0”.
(4) Family economic strain: Evaluated by a 4-item questionnaire adapted from Wadsworth and Compas (2002), concentrating on difficulties with covering expenses related to clothing, food, housing, and transportation. Participants responded on a scale from 1 (never) to 5 (always), and the reliability of the scale was strong, as indicated by a Cronbach’s α of 0.91. CFA demonstrated that the one-factor model exhibited a good fit to the data: χ^2^/df = 4.224, CFI = 0.91, TLI = 0.90, SRMR = 0.07, RMSEA = 0.08, and RMSEA 90% CI = [0.06, 0.09].
(5) Family cohesion: Assessed by the cohesion subscale of the Family Adaptability and Cohesion Evaluation Scale (FACES II) (Olson et al., 1983). In this study, to ensure consistency, the scoring was reversed, with higher scores indicating lower levels of cohesion, subsequently categorized as ‘low family cohesion.’ This 10-item instrument was rated on a 5-point scale ranging from 1 (not at all) to 5 (always). The Cronbach’s α for this study was 0.88. CFA demonstrated that the one-factor model exhibited a good fit to the data: χ^2^/df = 4.04, CFI = 0.94, TLI = 0.94, SRMR = 0.07, RMSEA = 0.07, and RMSEA 90% CI = [0.05, 0.08].
(6) Parent–child cohesion: The evaluation was conducted using a 20-item questionnaire adapted from W. Zhang et al. (2006). The questionnaire comprises two subscales: paternal cohesion and maternal cohesion. A low score on a single subscale is scored as 1 point, indicating the presence of one family risk factor, while a low score on both subscales is scored as 2 points, indicating two risk factors. Responses were rated on a 5-point scale ranging from 1 (not at all) to 5 (always). The Cronbach’s α for this study was 0.92. CFA showed that the two-factor model demonstrated an acceptable fit to the data. While the χ^2^/df ratio was elevated, other key indices supported the model’s adequacy (CFI = 0.93, TLI = 0.92, SRMR = 0.08, RMSEA = 0.08, and RMSEA 90% CI = [0.07, 0.09]).

The observed CFR range, distribution, and item prevalence are presented in Table 1.

#### 2.2.2. Relative Deprivation

The relative deprivation of adolescents was measured using the Relative Deprivation Scale developed by Ma (2012). This 4-item instrument assesses adolescents’ feelings and experiences regarding their perception of relative deprivation (e.g., “*I often feel that others have obtained what rightfully belongs to me*”). Each item is rated on a 6-point scale, ranging from 1 (strongly disagree) to 6 (strongly agree). This scale has demonstrated good reliability and validity in previous studies with Chinese children (Xie et al., 2025). The Cronbach’s α for this study was 0.78. CFA demonstrated that the one-factor model exhibited a good fit to the data: χ^2^/df = 2.54, CFI = 0.99, TLI = 0.99, SRMR = 0.01, RMSEA = 0.03, and RMSEA 90% CI = [0.00, 0.05].

#### 2.2.3. School Adjustment

School adjustment was assessed using the School Adjustment Scale developed by Cui (C2008). This 27-item instrument evaluates adolescent school adjustment across several dimensions, including academic adjustment (e.g., “*I often find myself distracted while studying*”), emotional and attitudinal responses to school (e.g., “*I hate going to school*”), peer relationships (e.g., “My classmates don’t like me”), teacher–student relationships (e.g., “*I am very afraid of my teachers*”), and routine adjustment (e.g., “*Some of the school rules make me feel uncomfortable*”). The scale has demonstrated good reliability and validity in previous studies with Chinese children (T. Tang et al., 2024). Each item is rated on a 5-point scale, ranging from 1 (strongly disagree) to 5 (strongly agree). The Cronbach’s α for this study was 0.90. CFA that the five-factor model exhibited a good fit to the data: χ^2^/df = 4.53, CFI = 0.95, TLI = 0.94, SRMR = 0.04, RMSEA = 0.05, and RMSEA 90% CI = [0.03, 0.06].

#### 2.2.4. Beliefs About Adversity

Beliefs about adversity were assessed using the Beliefs About Adversity Scale developed by Shek (2005). This 9-item instrument evaluates adolescents’ positive beliefs when confronting adversity (e.g., “*where there is a will, there is a way*”). Each item is rated on a 6-point scale, ranging from 1 (strongly disagree) to 6 (strongly agree). This scale has demonstrated good reliability and validity in previous studies with Chinese children (Liu & Wang, 2018). The Cronbach’s α for this study was 0.85. CFA demonstrated that the one-factor model exhibited a good fit to the data: χ^2^/df = 2.33, CFI = 0.96, TLI = 0.95, SRMR = 0.06, RMSEA = 0.07, and RMSEA 90% CI = [0.05, 0.08].

### 2.3. Procedure

The present study received approval from our university’s review board. Prior to the official research, verbal consent was obtained from the school administrators, educators, and students. The questionnaire survey took place in three urban schools, with all facilitators being psychology teachers and graduate students specializing in psychology. A group survey format was utilized for the participants, emphasizing voluntary participation, data confidentiality, and student anonymity. Participants were informed that they needed to respond independently based on their current circumstances.

### 2.4. Data Analysis

The statistical analyses were performed using SPSS 27.0 and Mplus 8.4. The data analysis proceeded according to the following steps: (1) Common method bias was examined using both Harman’s single-factor test and the unmeasured latent method factor (ULMC) approach. (2) Intraclass correlation coefficients (ICCs) were calculated to rule out potential clustering effects that might affect the robustness of the findings. (3) A series of preliminary analyses were conducted in SPSS 27.0, including descriptive statistics and bivariate correlations. Given the mixed data types, point-biserial correlation coefficients were computed for relationships involving binary variables. (4) Structural equation modeling was employed to test the direct effect of cumulative family risk on school adjustment among migrant children, as well as the mediating role of relative deprivation and the moderating role of beliefs about adversity. Model fit was evaluated using multiple indices including TLI, CFI, RMSEA, SRMR, and χ^2^/df.

## 3. Results

### 3.1. Common Method Bias

To mitigate common method bias, anonymous assessments and partially reversed scoring for some questions were implemented to regulate the testing procedure. In the analysis phase, Harman’s single-factor test was applied. The findings reveal that there were 27 factors with eigenvalues exceeding one, with the initial principal factor accounting for 24.68% of the variance (less than 40%), indicating that the study does not exhibit significant common method bias.

However, given the limited sensitivity of Harman’s test (D. Tang & Wen, 2020), we further applied the unmeasured latent method factor (ULMC) approach to more rigorously evaluate common method bias. The analysis incorporated six key constructs: family economic strain, family cohesion, parent–child cohesion, relative deprivation, school adjustment, and beliefs about adversity. After introducing a common method factor into the original six-factor model, model fit was not significantly improved: χ^2^ = 5963.08, *p* < 0.01; CFI = 0.93; TLI = 0.95; RMSEA = 0.07; and SRMR = 0.06. The changes in fit indices were negligible (ΔCFI = 0.02, ΔTLI = 0.00, ΔRMSEA = 0.01, and ΔSRMR = 0.01). Thus, it can be concluded that common method bias does not pose a serious threat in this study.

### 3.2. Assessment of Clustering Effects

To address potential clustering effects arising from the recruitment of participants from three secondary schools, we computed intraclass correlation coefficients (ICCs) for the key variables—relative deprivation, beliefs about adversity, and school adjustment—before estimating the moderated mediation model. The obtained ICC values were 0.039 (95% CI: [−0.05, 0.13]) for relative deprivation, 0.045 (95% CI: [−0.04, 0.14]) for beliefs about adversity, and 0.001 (95% CI: [−0.08, 0.09]) for school adjustment. All confidence intervals included zero, confirming that school-level clustering was negligible in this study.

### 3.3. Preliminary Analyses

Table 2 presents the descriptive statistics, including means, standard deviations, and correlations among the primary study variables. Cumulative family risk exhibited a significant positive correlation with relative deprivation, while showing a significant negative correlation with both school adjustment and beliefs about adversity. Furthermore, relative deprivation was significantly negatively correlated with both school adjustment and beliefs about adversity. In contrast, school adjustment demonstrated a significant positive correlation with beliefs about adversity.

### 3.4. Testing for the Mediating Role of Relative Deprivation

First, a robustness check was conducted by estimating the structural equation model (using maximum likelihood) with two operationalizations of cumulative family risk: the dichotomous 75th percentile measure and a continuous standardized composite. The key findings were consistent across both specifications. The model exhibited excellent fit: χ^2^/df = 3.28, TLI = 0.97, CFI = 0.99, RMSEA = 0.065, and SRMR = 0.014. Controlling for gender and age, a positive path was established from cumulative family risk to relative deprivation (β = 0.28 and *p* < 0.001), and a negative path from relative deprivation to school adjustment (β = −0.15 and *p* < 0.001; see Table 3). The residual direct effect was also significant (β = −0.13, *p* < 0.001, and bootstrap 95% CI [−0.16, −0.10]), indicating partial mediation. This was supported by a significant indirect effect (indirect effect = −0.04 and bootstrap 95% CI [−0.07, −0.01]; see Table 4), which accounted for 23.53% of the total effect.

### 3.5. Testing for Moderated Mediation

**Figure 2:**
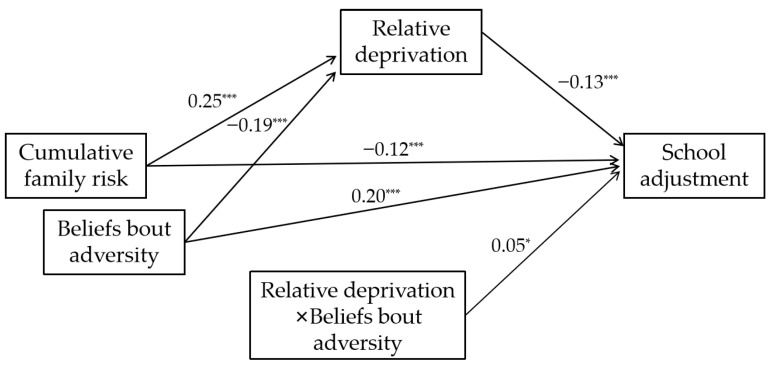
Moderated mediation model. *** *p* < 0.001 and * *p* < 0.05.

Next, a model was tested with beliefs about adversity serving as the moderator between relative deprivation and school adjustment. The model demonstrated an excellent fit to the data: χ^2^/df = 3.95, CFI = 0.99, TLI = 0.97, RMSEA = 0.032, and SRMR = 0.013. As shown in Table 5 and Figure 2, after controlling for gender and age, the interaction term between RD and BAs significantly predicted school adjustment (β = 0.05, *p* < 0.05, bootstrap 95% CI [0.01, 0.08]).

**Figure 3:**
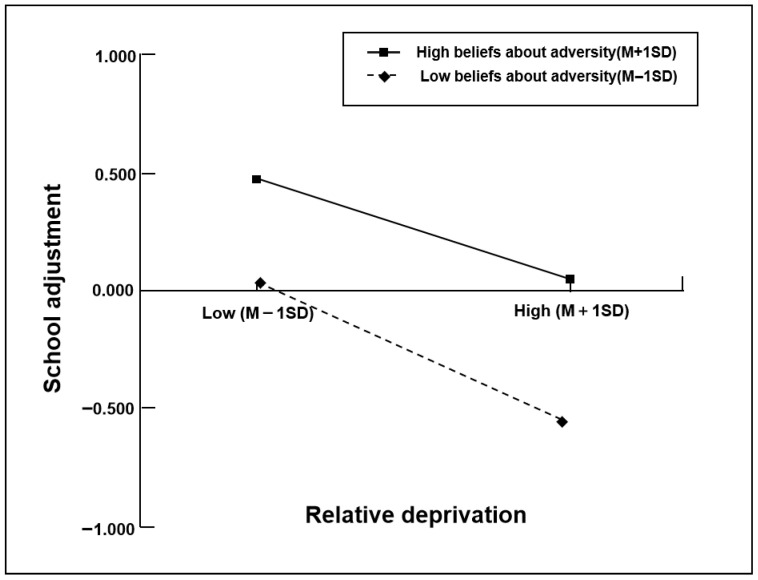
Beliefs about adversity moderate the effect of relative deprivation on school adjustment.

The simple slopes analysis revealed that, for migrant children with weaker beliefs about adversity (M − 1SD), higher levels of relative deprivation were associated with lower levels of school adjustment (βsimple = −0.31, *p* < 0.001). Conversely, for migrant children with stronger beliefs about adversity (M + 1SD), the effect of relative deprivation on school adjustment was significantly weaker (βsimple = −0.20, *p* < 0.001) (see Figure 3). Thus, relative deprivation emerged as a much stronger predictor of school adjustment for migrant children with lower levels of beliefs about adversity, aligning with the stress-buffering model.

## 4. Discussion

The current study provides evidence that the negative association between cumulative family risk and school adjustment is mediated by relative deprivation, and that this direct effect is moderated by beliefs about adversity. The study’s major contribution is that it deepens our understanding of how cumulative family risk is associated with migrant children’s school adjustment. Furthermore, the complex moderated mediation model offers insights useful for potential prevention and intervention programs aimed at reducing problems migrant children may face regarding their school adjustment.

Consistent with hypothesis H1, cumulative family risk demonstrates a significant association with school adjustment among migrant children. This result suggests that cumulative family risk serves as a critical risk factor influencing externalizing problem behaviors in migrant children, aligning with previous research and reinforcing the cumulative risk model (Evans et al., 2013; Kliewer et al., 2017). As noted by Evans et al., the presence of multiple simultaneous risk factors greatly heightens the chances of negative developmental results in children (Evans et al., 2013). This effect is particularly salient among middle school-aged migrant children, a population undergoing a critical developmental transition. Within urban education systems, these children constitute a vulnerable group, confronting not only typical school adjustment challenges but also compounding socio-environmental stressors. When such risk factors accumulate and interact, their synergistic effects can significantly exacerbate difficulties in the school adjustment process.

Consistent with hypothesis H2, the findings reveal that relative deprivation plays a partial mediating role in the relationship between cumulative family risk and the school adjustment of migrant children. The results indicate that cumulative family risk affects school adjustment through both direct pathways and those mediated by relative deprivation.

The results clearly indicate that an accumulation of family-related risks greatly heightens feelings of relative deprivation, which is consistent with earlier research and supports the theory of parental relative deprivation (Pettigrew, 2016; Smith et al., 2012). Prolonged exposure to various family risks results in negative self-assessments and a sense of unfair disadvantage, leading to feelings of relative deprivation (Pettigrew, 2016; J. Zhang et al., 2022). Importantly, our findings regarding migrant children reflect established research, indicating that multiple family risk factors reliably predict the development of relative deprivation (Pinchak & Swisher, 2022). Additionally, the research shows that relative deprivation has a significant negative impact on the school adjustment of migrant children, aligning with previous studies (Keshavarz et al., 2021; Mishra & Carleton, 2015). This detrimental psychological state fosters perceptions of disadvantage through social comparisons, adversely affecting various domains of school adjustment. These results underscore the dual nature of adjustment mechanisms in novel educational settings; while cumulative risk factors remain crucial, equal consideration must be given to the mediating role of subjective psychological experiences in shaping adjustment outcomes.

Consistent with hypothesis H3, the moderation analysis indicates that perceptions of adversity play a significant role in influencing the connection between relative deprivation and school adjustment. Analysis of the data shows that as migrant children’s perceptions of adversity rise, the detrimental effect of relative deprivation on their school adjustment notably diminishes, which aligns with earlier research and cognitive adaptation theory (Keshavarz et al., 2021). As a beneficial psychological asset, beliefs regarding adversity bolster migrant children’s optimism and psychological resilience (Mou et al., 2013), strengthen their belief in being masters of their own destiny, and enable them to actively seek value and meaning in adversity (Q. Wang & Liu, 2023; Zhao et al., 2017). This transformation of unfavorable circumstances into motivation for personal development assists their adjustment to new school environments (Shek, 2004; Zhao et al., 2013).

Based on the cumulative risk model, relative deprivation theory, and cognitive adaptation theory, this research develops a moderated mediation framework that clarifies how cumulative family risk affects the school adaptation of migrant children. By merging theoretical insights with empirical data, this study enhances the comprehension of the school adaptation journey for migrant youth and offers vital theoretical and empirical support for educational interventions. Our findings indicate that cumulative family risk has a direct and adverse impact on the school adaptation of migrant children but also affects this process indirectly through the mediating influence of relative deprivation. This insight has important practical ramifications: when creating interventions for migrant children facing high risks, it is essential not only to improve their family circumstances but also to implement psychological strategies that alleviate their feelings of relative deprivation, thereby promoting better school adaptation. Future intervention strategies should therefore employ a dual approach that tackles both the reduction of objective risk factors and the management of subjective feelings of deprivation. Additionally, the examination of moderating effects underscores the significance of nurturing positive belief about adversity among migrant children. While the protective effect of beliefs about may lessen in the presence of multiple overlapping risks, it still serves to mitigate the negative consequences on school adaptation stemming from perceived disadvantages. This indicates that systematic training in beliefs about adversity can help reshape individuals’ negative views of their disadvantaged circumstances, thus lessening the consequent detrimental effects on school adaptation. Therefore, when executing focused mental health education, it is crucial for schools and educators to weave the development of beliefs about adversity into the educational framework. Through methods such as cognitive restructuring, situational role-playing, and resilience training, students can cultivate positive perspectives on adversity and strengthen their psychological resilience, better preparing them to navigate challenges and pressures in their environments.

This research explores how cumulative family risk impacts school adjustment in migrant children, emphasizing the mediating influence of relative deprivation and the moderating effect of beliefs about adversity. The results offer valuable insights that can prove useful for the creation of prevention and intervention programs tailored to high-risk migrant children struggling with school adaptation. Nonetheless, three key limitations must be noted: Firstly, the cross-sectional nature of the study restricts our ability to analyze age-related differences in the influence of cumulative family risk; longitudinal research in the future could more effectively track these effects over time. Secondly, although the seven identified family risk factors are significant, future studies might expand their focus to include additional family risks (such as parental neglect) and ecological risks from educational and community environments, leading to a more thorough evaluation of cumulative risk impacts. Lastly, the assessment of four risk factors relied on the participants’ relative position within the sample, which could create sample-specific biases—a common methodological issue in cumulative risk research (Appleyard et al., 2005). Therefore, caution is warranted when applying these findings broadly due to potential concerns regarding sample homogeneity.

## 5. Conclusions

Empirical evidence confirms that cumulative family risk not only directly and negatively predicts migrant children’s school adjustment, but also indirectly influences their social adaptation through relative deprivation, with beliefs about adversity moderating the latter part of this mediating pathway. The present study not only deepens the theoretical understanding of the relationship between cumulative family risk and school adjustment and its underlying mechanisms, but also provides practical insights useful for the design of interventions that aim to enhance school adjustment among migrant children.

## Figures and Tables

**Table 1:** Prevalence of Cumulative Family Risk Factors and Score Distribution among Migrant Children (N = 1128).

CFR Factor	Migrant Children							
	N				%			
Family structure	333				29.52			
Educational attainment of parents	998				88.48			
Parent–child separation	743				65.87			
Family economic strain	282				25.00			
Family cohesion	282				25.00			
Paternal cohesion	282				25.00			
Maternal cohesion	282				25.00			
CFR range	0	1	2	3	4	5	6	7
Distribution (*n*)	245	360	194	135	126	61	6	1
Proportion (%)	21.72	31.91	17.20	11.97	11.17	5.41	0.53	0.09

**Table 2:** Means, standard deviations, and intercorrelations of study variables (N = 1128).

Variables	M ± SD	1	2	3	4	5	6
1. Gender	_	1					
2. Age	12.83 ± 1.21	0.02	1				
3. Cumulative family risk	1.78 ± 1.52	0.01	0.03	1			
4. Relative deprivation	2.32 ± 1.19	−0.04	−0.03	0.36 **	1		
5. Beliefs about adversity	4.40 ± 0.80	−0.10 *	−0.04	−0.37 **	−0.23 **	1	
6. School adjustment	4.14 ± 0.64	0.00	−0.07 *	−0.52 **	−0.43 **	0.44 **	1

Notes: Statistical significance is denoted by * *p* < 0.05 and ** *p* < 0.01.

**Table 3:** Mediation effects of relative deprivation on the relationship between cumulative family risk and school adjustment.

Variables	Relative Deprivation		School Adjustment	
	β	*SE*	β	*SE*
Gender	−0.03	0.06	−0.03	0.01
Age	−0.07	0.03	−0.05	0.03
CFR	0.28	0.04	−0.13	0.02
RD			−0.15	0.02

**Table 4:** The bootstrapping analysis of the mediating effects.

	Effect	*SE*	95% CI	Proportion (%)
Total effect	−0.17	0.01	[−0.19, −0.08]	100%
Direct effect	−0.13	0.01	[−0.16, −0.10]	76.47%
Indirect effect	−0.04	0.01	[−0.07, −0.01]	23.53%

**Table 5:** Results of beliefs about adversity moderate the mediation process.

Variables	Relative Deprivation			School Adjustment		
	β	*SE*	95% CI	β	*SE*	95% CI
Gender	−0.02	0.05	[−0.12, 0.08]	0.03	0.03	[−0.03, 0.08]
Age	−0.06	0.06	[−0.18, 0.07]	0.02	0.03	[−0.05, 0.06]
CFR	0.25	0.05	[0.12, 0.28]	−0.12	0.02	[−0.15, −0.08]
BA	−0.19	0.04	[−0.27, −0.11]	0.20	0.02	[0.11, 0.27]
RD				−0.13	0.01	[−0.15, −0.06]
RD × BA				0.05	0.01	[0.01, 0.08]

Note: β is the standardized regression coefficient.

## Data Availability

The raw data supporting the conclusions of this article will be made available by the authors on request.
